# Supplementary material for: Cross-Linked Alginate Dialdehyde/Chitosan Hydrogel Encompassing Curcumin-Loaded Bilosomes for Enhanced Wound Healing Activity
Source: Pharmaceutics. 2024 Jan 9;16(1):90. doi: 10.3390/pharmaceutics16010090 (PMC10819348; doi:10.3390/pharmaceutics16010090)
Supplement: Supplementary file 1 [file pharmaceutics-16-00090-s001.zip › pharmaceutics-2738294-supplementary.pdf]

Supplementary file

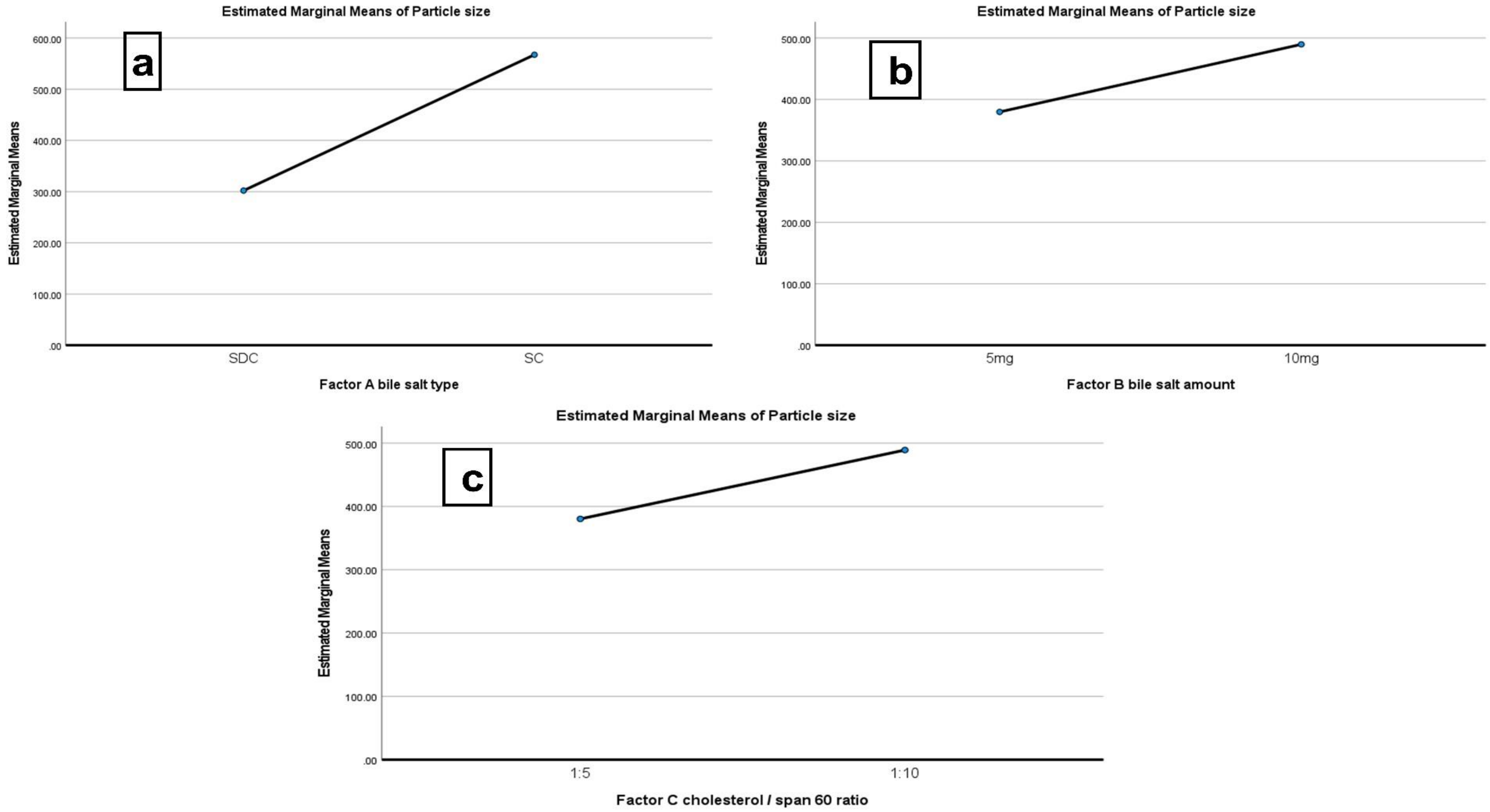

Figure S1. Line graphs showing the effect of: (a) Type of bile salt, (b) Amount of bile salt, and (c) Cholesterol /Span® 60 ratio on particle size.

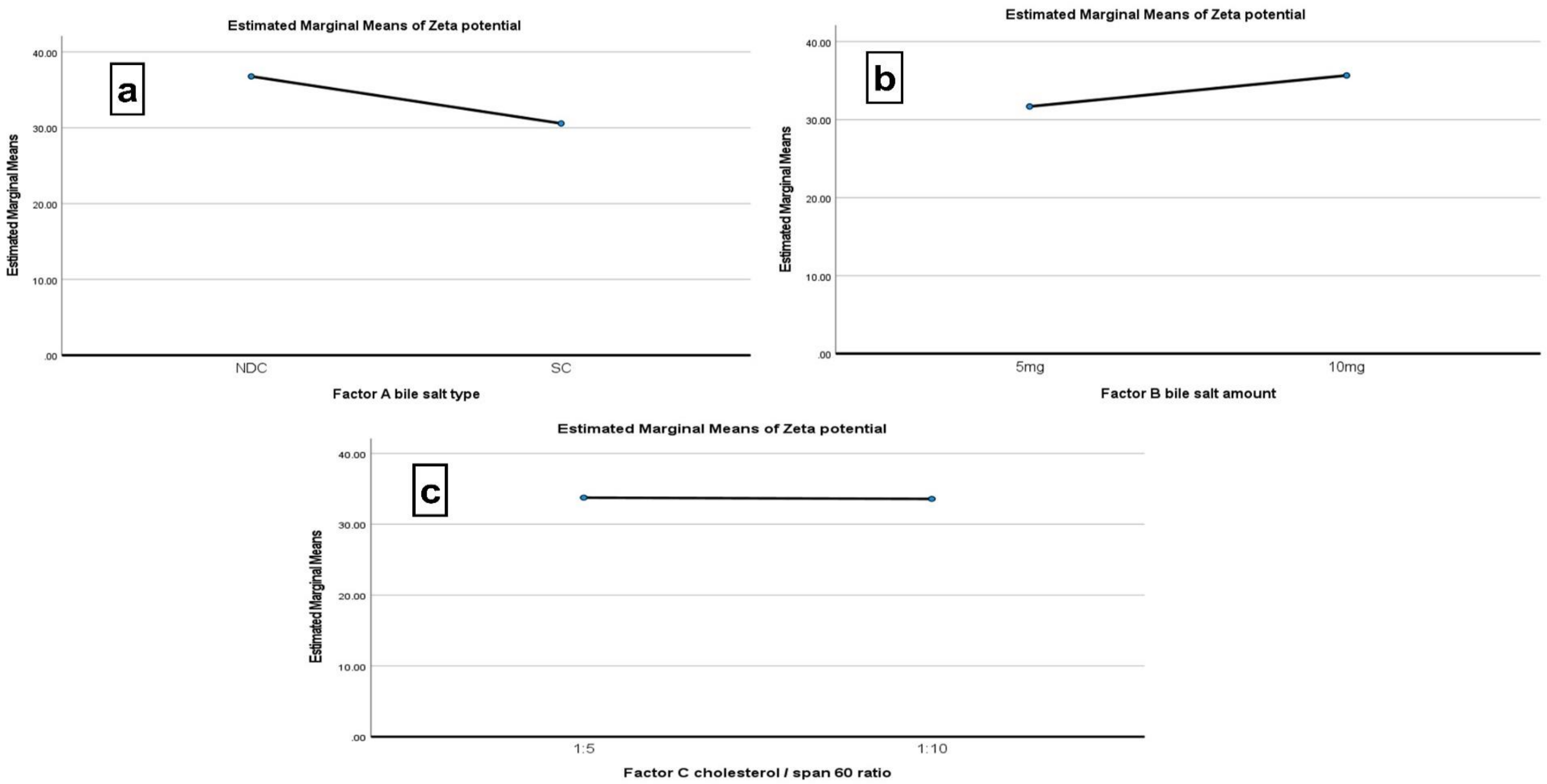

Figure S2. Line graphs for the effect of: (a) Type of bile salt, (b) Amount of bile salt, and (c) Cholesterol /Span® 60 ratio on zeta potential.

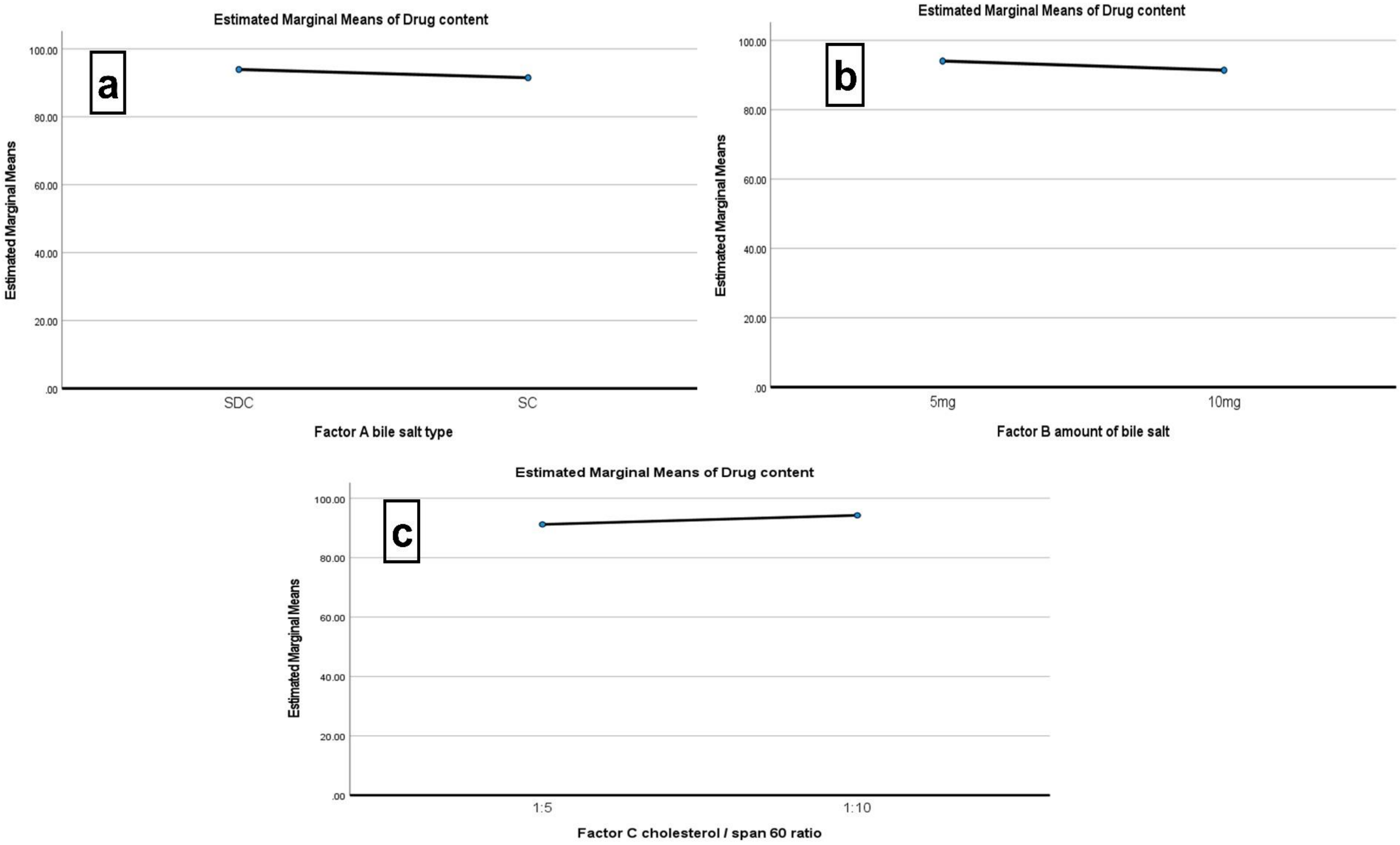

Figure S3. Line graphs for the effect of: (a) Type of bile salt, (b) Amount of bile salt, and (c) Cholesterol /Span® 60 ratio on drug content.

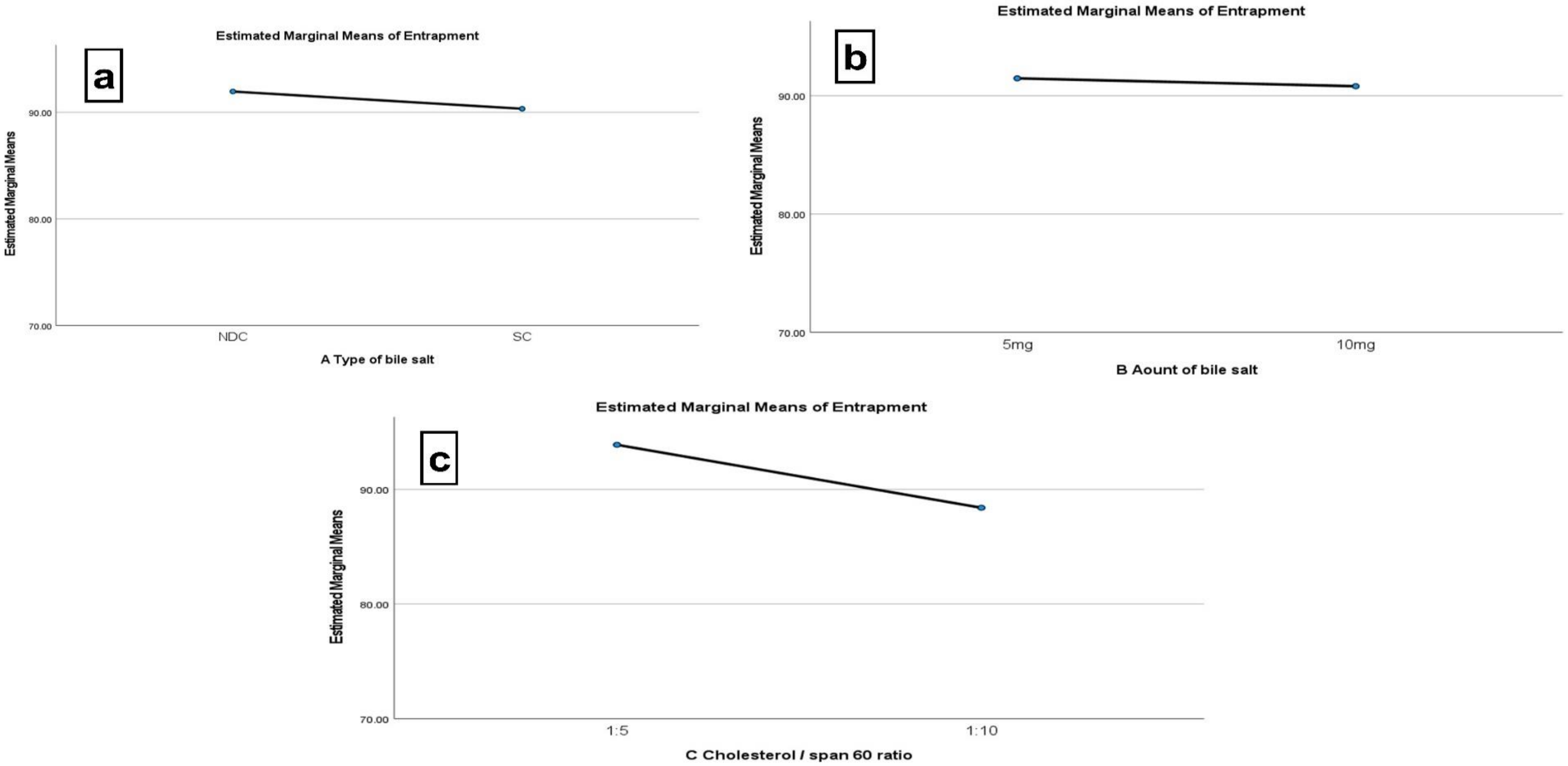

Figure S4. Line graphs for the effect of: (a) Type of bile salt, (b) Amount of bile salt, and (c) Cholesterol /Span® 60 ratio on entrapment efficiency (%).

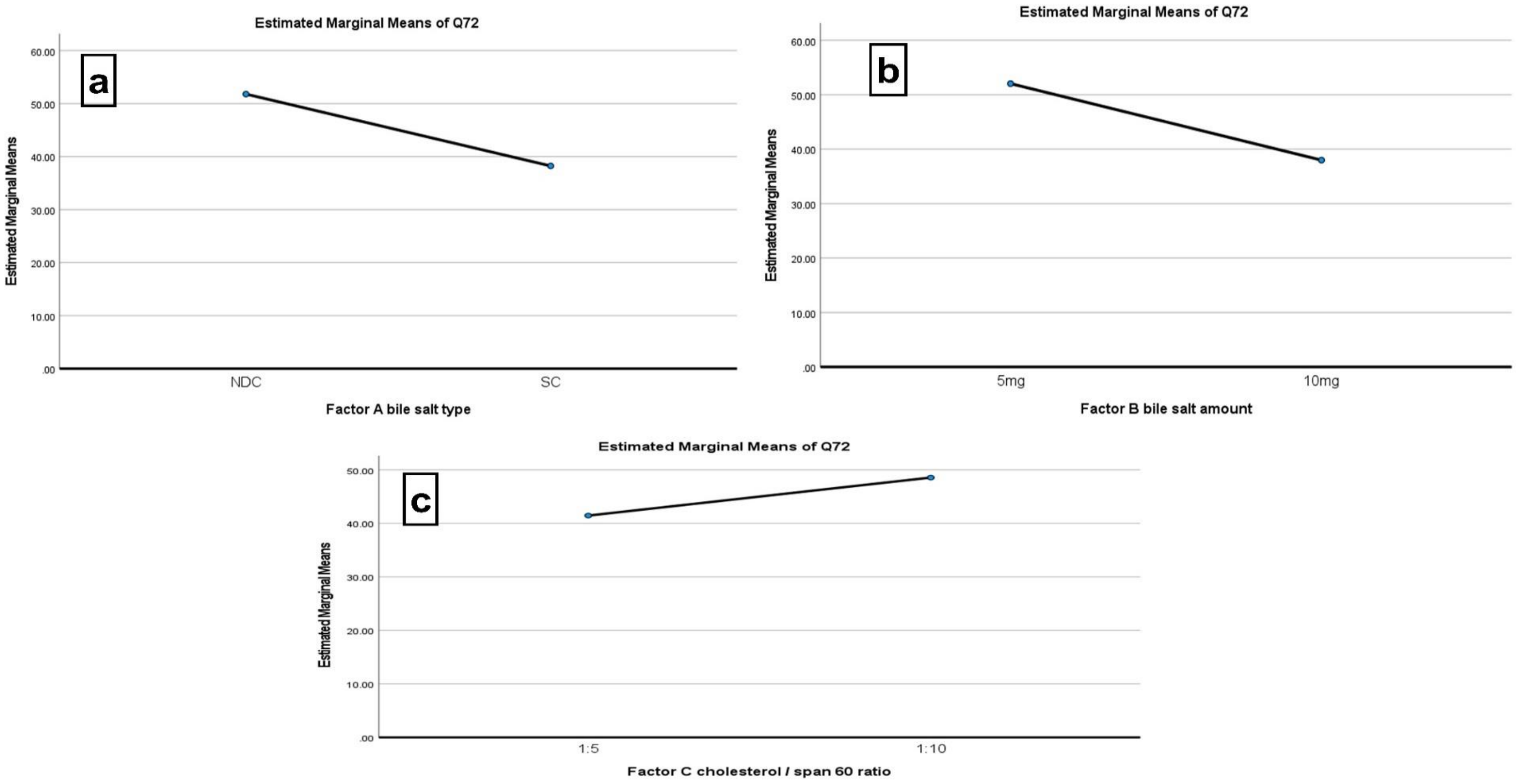

Figure S5. Line graphs for the effect of: (a) Type of bile salt, (b) Amount of bile salt, and (c) cholesterol /Span® 60 ratio on % drug released after 72 h (Q_{72h}).

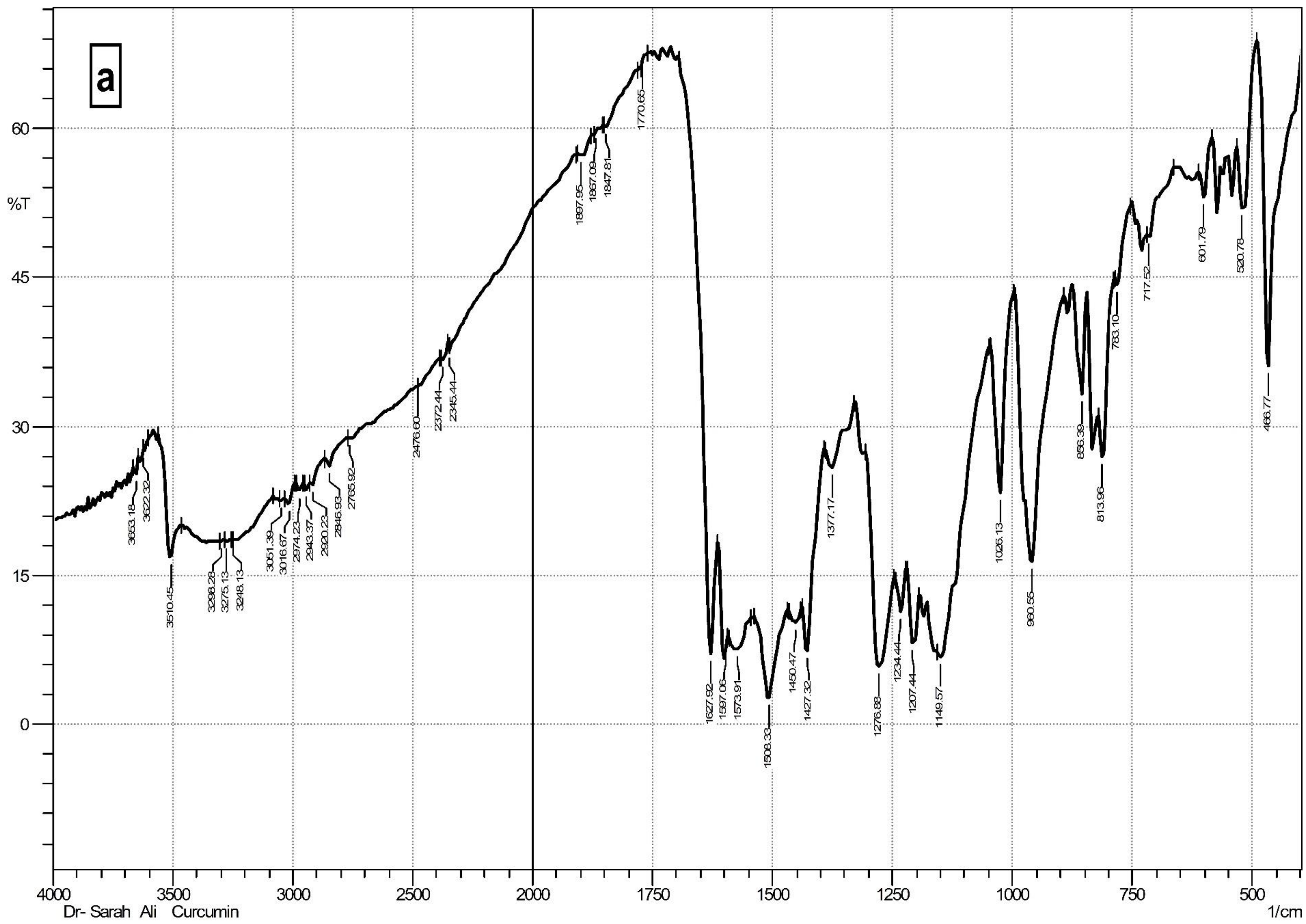
a
%T
60
45
30
15
0
4000
3500
3000
2500
2000
1750
1500
1250
1000
750
500
1/cm
Dr- Sarah Ali Curcumin
3653.18
3622.32
3510.45
3298.28
3275.13
3248.13
3051.39
3016.67
2974.23
2943.37
2920.23
2846.93
2765.92
2476.60
2372.44
2345.44
1897.95
1867.09
1847.81
1770.65
1627.92
1597.06
1573.91
1508.33
1450.47
1427.32
1377.17
1276.88
1234.44
1207.44
1149.57
1026.13
960.55
856.39
813.96
783.10
717.52
601.79
520.78
466.77

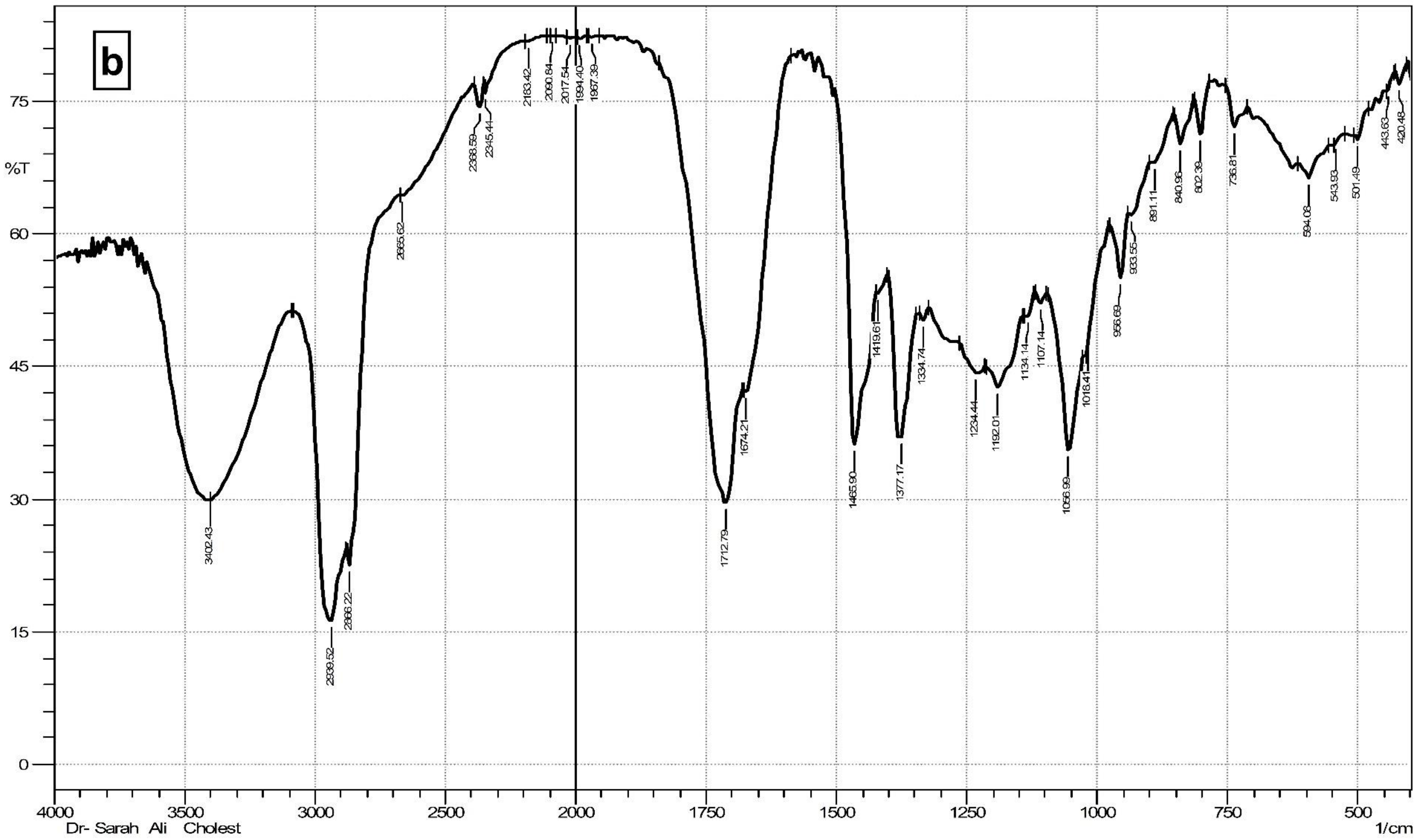
b
%T
75
60
45
30
15
0
3402.43
2939.52
2866.22
2665.62
2368.59
2345.41
2183.42
2090.84
2017.54
1994.40
1967.39
1712.79
1674.21
1465.90
1419.61
1377.17
1334.74
1234.44
1192.01
1134.14
1107.14
1056.99
1018.41
956.69
933.55
891.11
840.96
802.39
736.81
594.08
543.93
501.49
443.63
420.48
4000
3500
3000
2500
2000
1750
1500
1250
1000
750
500
1/cm
Dr- Sarah Ali Cholest

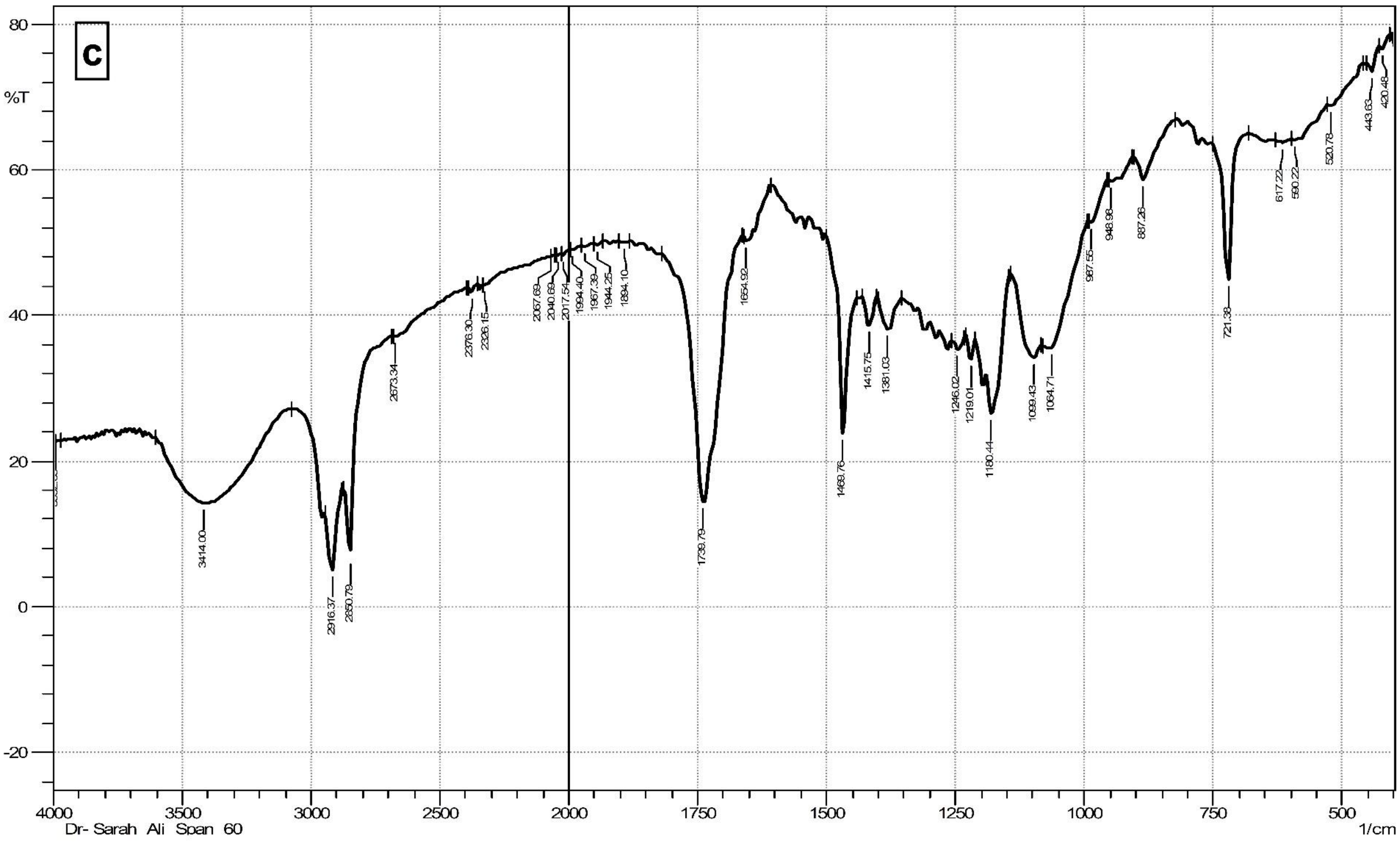
c
%T
80
60
40
20
0
-20
4000
3500
3000
2500
2000
1750
1500
1250
1000
750
500
1/cm
Dr- Sarah Ali Span 60
3414.00
2916.37
2850.79
2673.34
2376.30
2326.15
2067.69
2040.69
2017.54
1994.40
1967.39
1944.25
1894.10
1739.79
1654.92
1469.76
1415.75
1381.03
1246.02
1219.01
1180.44
1099.43
1064.71
987.55
948.98
887.26
721.38
617.22
590.22
520.78
443.63
420.48

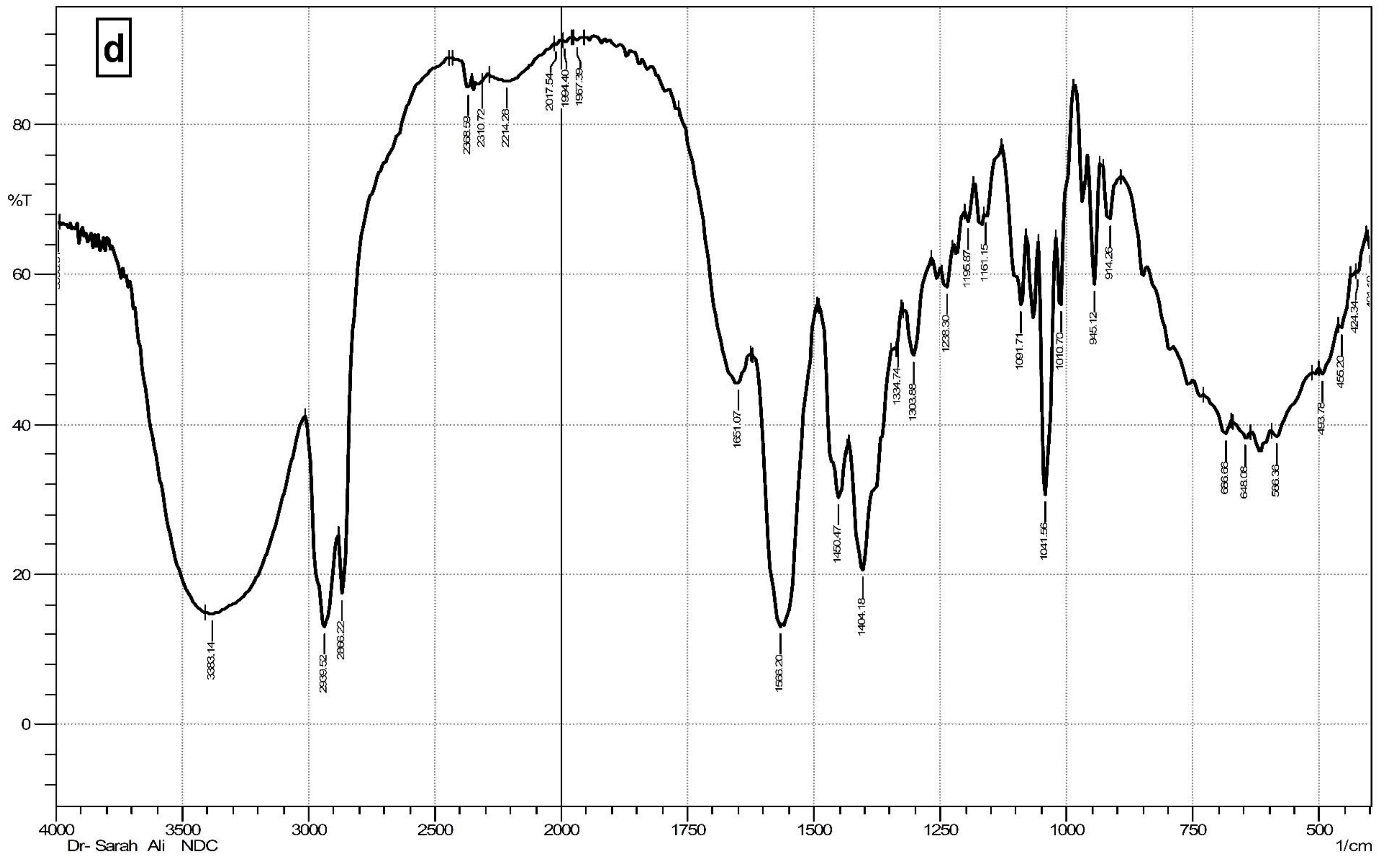
d
%T
1/cm
Dr- Sarah Ali NDC
3383.14
2939.52
2866.22
2368.59
2310.72
2214.28
2017.54
1994.40
1967.39
1651.07
1566.20
1450.47
1404.18
1334.74
1303.88
1238.30
1195.87
1161.15
1091.71
1041.56
1010.70
945.12
914.26
686.66
648.08
586.36
493.78
455.20
424.34

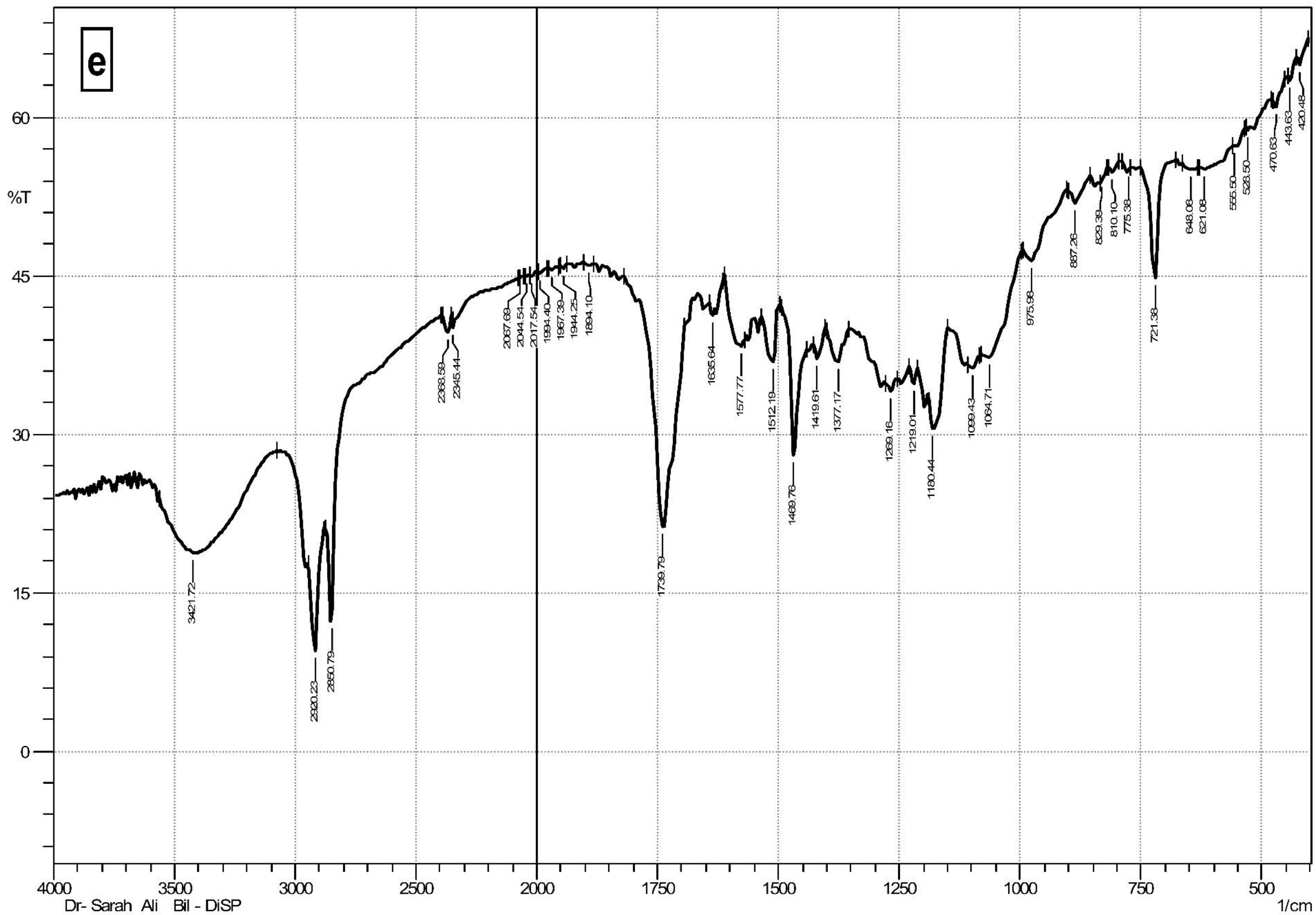

e
%T
60
45
30
15
0
3421.72
2920.23
2850.79
2368.59
2345.44
2067.69
2044.54
2017.54
1994.40
1967.39
1944.25
1894.10
1739.79
1635.64
1577.77
1512.19
1469.76
1419.61
1377.17
1269.16
1219.01
1180.44
1099.43
1064.71
975.98
887.26
829.39
810.10
775.38
721.38
648.08
621.08
555.50
528.50
470.63
443.63
420.48
4000
3500
3000
2500
2000
1750
1500
1250
1000
750
500
1/cm
Dr- Sarah Ali Bil - DiSP

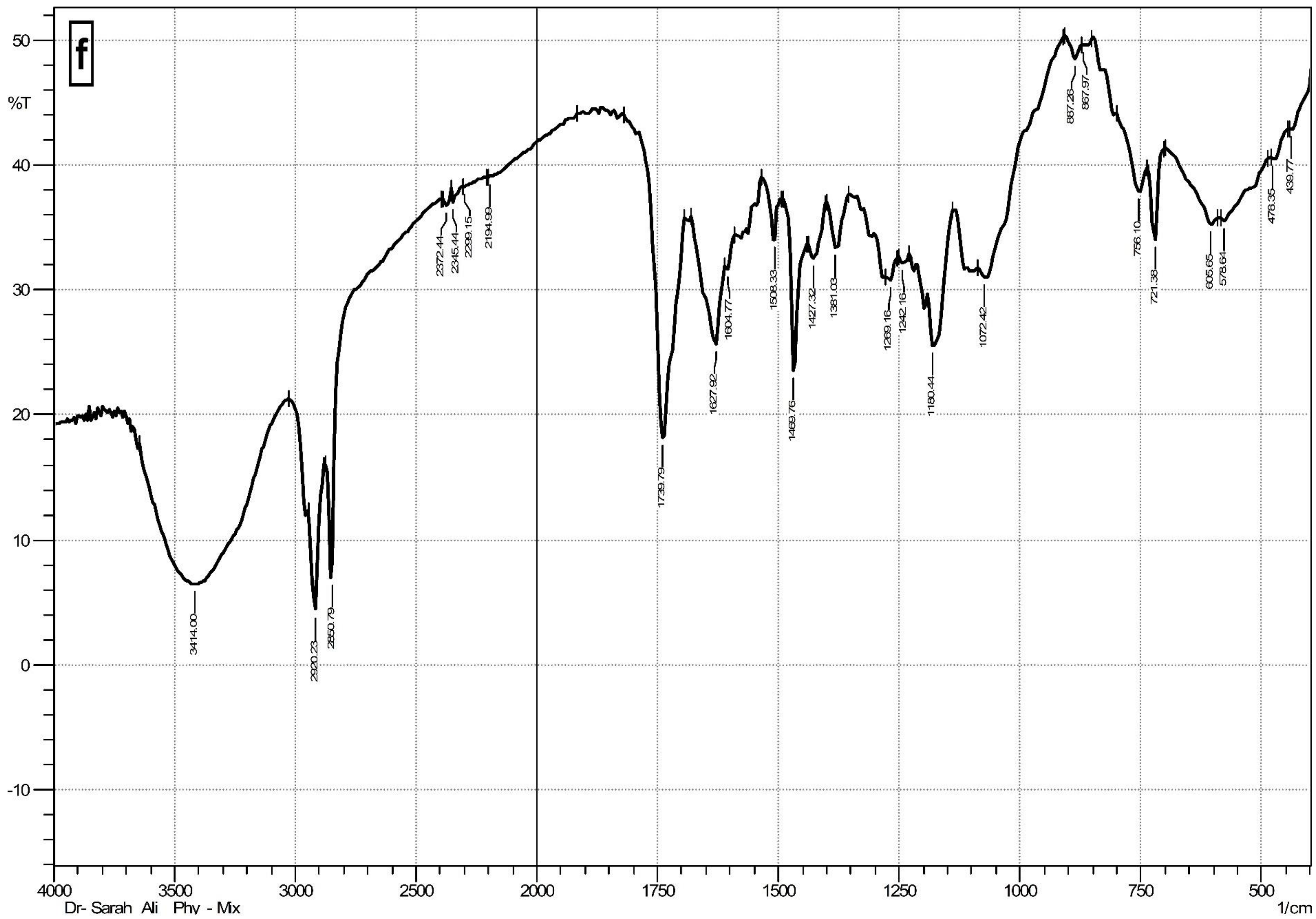
f
%T
50
40
30
20
10
0
-10
3414.00
2920.23
2850.79
2372.44
2345.44
2299.15
2194.99
1739.79
1627.92
1604.77
1508.33
1469.76
1427.32
1381.03
1269.16
1242.16
1180.44
1072.42
887.26
867.97
756.10
721.38
605.65
578.64
478.35
439.77
4000
3500
3000
2500
2000
1750
1500
1250
1000
750
500
1/cm
Dr- Sarah Ali Phy - Mix

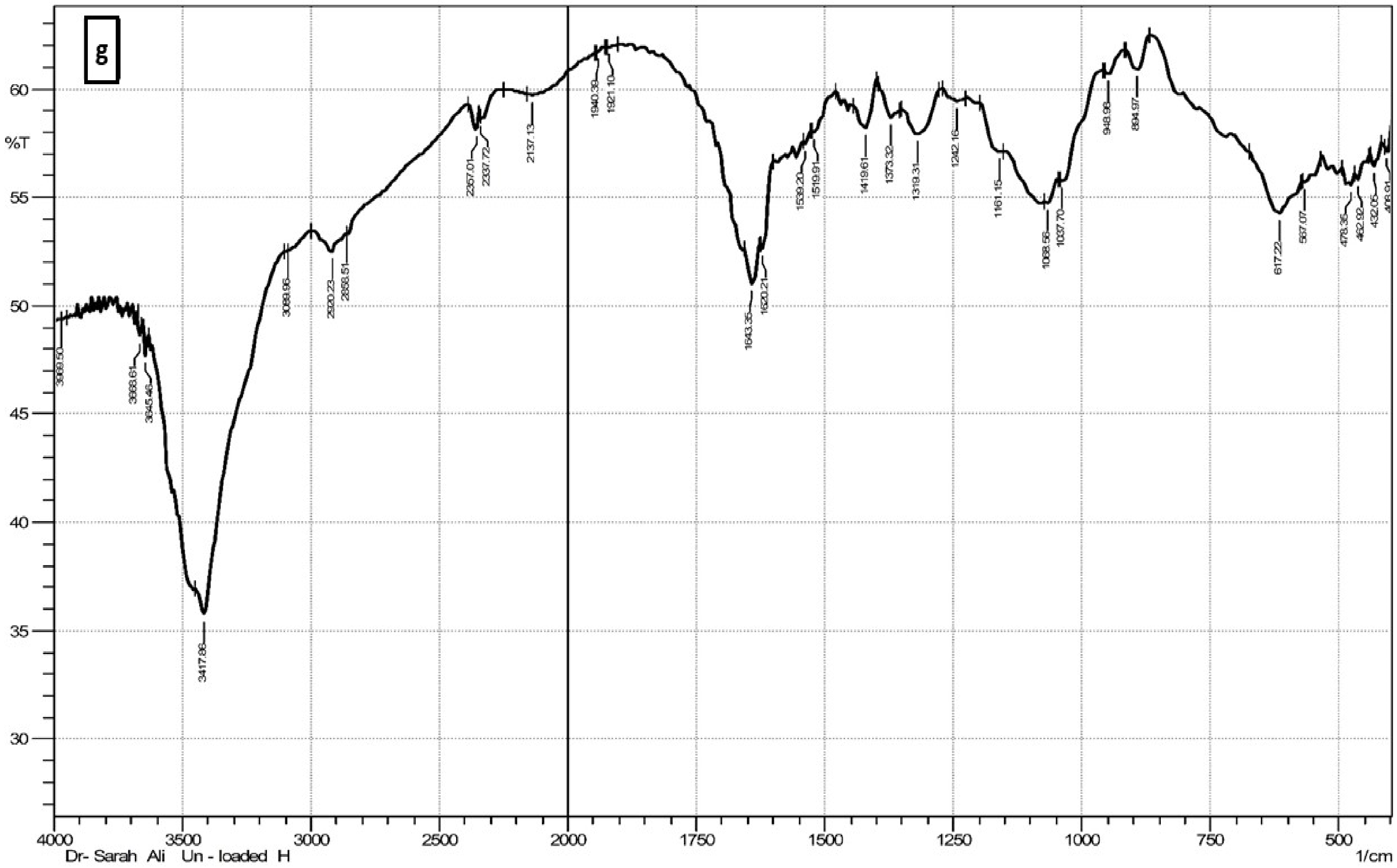

g
%T
60
55
50
45
40
35
30
4000
3500
3000
2500
2000
1750
1500
1250
1000
750
500
1/cm
Dr- Sarah Ali Un - loaded H
3417.86
1643.35
1620.21
1068.56
1037.70
617.22
2357.01
2337.72
2137.13
1940.39
1921.10
2920.23
2858.51
3089.96
1539.20
1519.91
1419.61
1373.32
1319.31
1242.16
1161.15
948.98
894.97
567.07
478.35
462.92
432.05
3645.46
3668.61
3969.50

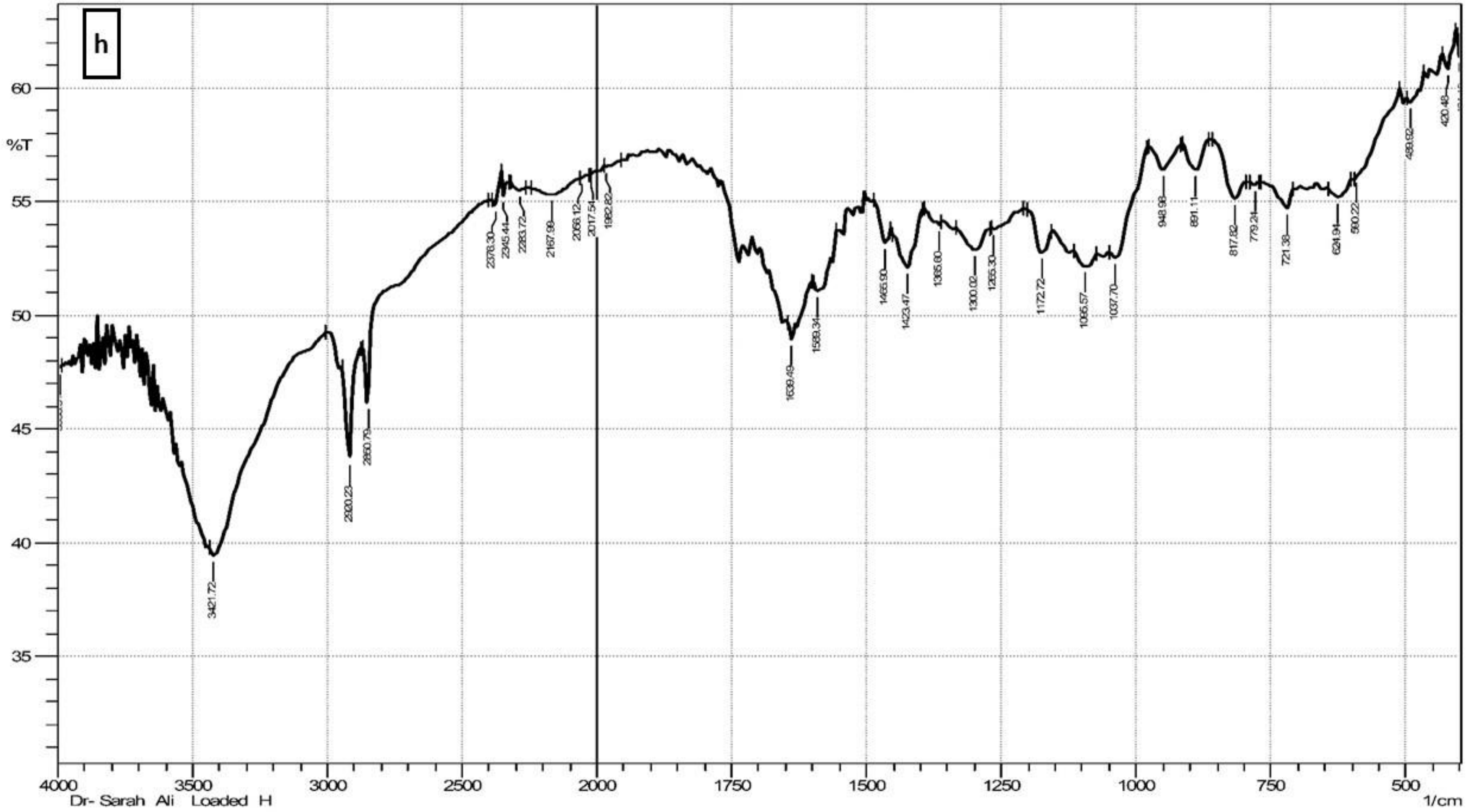

Figure S6. IR graphs of (a) curcumin, (b) cholesterol, (c) Span® 60, (d) sodium deoxycholate, (e) bilosomal formula F5 (as per mentioned in Table 1), (f) physical mixture, (g) plain and (h) curcumin loaded bilosomal hydrogel).
